# Notes and News

**Published:** 1952-07

**Authors:** 


### *Notes and News*

### SENIOR DEGREES AND DIPLOMAS

| | |
|---|---|
| *M.R.C.P.* (*Ed.*)<br>*M.R.C.P.* (*London*) | Dr. S. L. Balse. |
| *D.P.M.* (*London*) | Dr. B. V. Earle. |
| *D.C.H.* (*Dublin*). | Dr. H. N. Saran. |

### PRIZES

Dr. S. Smith, Assistant Lecturer in Psychiatry and Physician, Barrow Hospital, has been awarded the Gaskell Medal and Prize 1952 of the Royal Medico-Psychological Association.

Dr. E. H. Hare, Tutor in Psychiatry and Physician, Barrow Hospital, has been awarded the Bronze Medal and Prize of the R.M.P.A. for 1952.

*Dental Gold Medal* (*Final B.D.S., December*, 1951)—Mr. J. P. Fletcher.

*Thomas Francis Edgeworth* and *Francis Henry Edgeworth Prize*—Mr. D. H. Wright.

*Western Dental Mfg. Co. Prize*—Mr. K. J. Mullan.

### UNIVERSITY OF BRISTOL

*Degrees of M.B., Ch.B.* (*Brist.*)

*June* 1952

Appelbe, Frederick James

Arney, Kenneth Brian

Baggott, William Malcolm

Bailey, Helena Bozena Marie

Bennett, Bernard George

Brown, June Muriel

Brown, Stewart Harman

Cobb, Nigel John

Coles, Brian James Fitzwalter

Colston, Brian

Crook, Peter Austin

Duff, William John Peter

Edmondson, Kenneth William

Fawcett, Kenneth John Rhodes

Gawthorn, Edward Charles

(*Distinction in Obstetrics*)

Gear, Douglas John

Hamblett, Eric Percival

Higgins, Gerald Leslie

Hooper, Audrey Vera

Jackman, Michael Edward

(*Distinction in Obstetrics*)

Jenkins, John Ward

Jordan, Arthur Frederick

King, Marguerite Angela Ruth

Laxton, Pearl Desiree

Light, Joan Iris

Livesey, Brian

Lowther, Gordon Percy

Martyn, Sheila Ann

(*Distinction in Obstetrics*)

Otlet, Alan Benjamin

Prowse, Alison Winter

Sanerkin, Nedjdet Gurgen

(*2nd Class Hons.*)

Sellwood, Ronald Arthur

Walker, Andrew George

Wood, Geoffrey Holman

Worsley, David Eric

Professor R. J. Brocklehurst will give the Long Fox Memorial Lecture on October 21st, 1952, on "**Progress in Medical Education.**"

We announce with regret the death of Mr. W. STUART V. STOCK, who was a student of the Medical School, 1892–98, and Demonstrator in Anatomy in the University from 1909–35 and Lecturer in Charge of the Department of Anaesthetics and Hon. Anaesthetist, Bristol Royal Infirmary, from 1929 until his retirement in 1935.

### BRISTOL MEDICO-CHIRURGICAL SOCIETY

Dr. G. E. F. SUTTON, *M.C.*, Physician Bristol Royal United Hospitals and treasurer of the Society, has been elected President of the Bristol Medico-Chirurgical Society for the session 1952–53, on the resignation of Mr. E. WATSON-WILLIAMS.

### WESSEX RAHERE CLUB

The Autumn Dinner of the above Club will take place at the Grand Spa Hotel, Clifton, Bristol, on Saturday, October 25th.

It is hoped that, as usual, a member of the Staff will be present as Guest of Honour.

Membership of the Club is open to all Barts' men practising in the West Country.

Further details will be circulated to members and to any other Barts' men who are interested and who will get in touch with the Hon. Secretary, Mr. A. DAUNT BATEMAN of 3 The Circus, Bath.

